# New Rates for Army Nurses

**Published:** 1920-09-11

**Authors:** 


### NEW RATES FOR ARMY NURSES.

The new annual rates of pay of the Queen Alexandra Imperial Military Nursing Service, and of the permanent nursing establishment of the Military Families Hospitals, just issued as an Army Order, are as follows: For the Q.A.I.M.N.S.—Matron £115 minimum, rising by £10 yearly to a maximum of £185: assistant matron £85, rising by £5 a year to £95; sister £75, rising by £5 to £85; and staff nurse £60, rising by £2 10s. to £65. For the nursing staff of Military Families Hospitals: Matron at Aldershot £115, rising by £10 increments to £185 maximum; matron at other stations, £75 by £5 to £85; and charge nurse £60, by £2 10s. increments to £65. These rates have effect from April 1, 1920. In addition to these rates charge pay for matrons of Q.A.I.M.N.S will be at rates not exceeding £45 a year, and for matrons at Military Families Hospitals at stations other than Aldershot £20 or £30 a year according to the number of beds. Retired pay will comprise a service element based on total service (£3 for each year of service), and a rank element for the rank from which the nurse retires; but a nurse retiring with less than ten years' complete service will not be eligible for retired pay. The maximum rates of these two elements together are as follows: Matron Q.A.I.M.N.S. and at Military Families Hospital at Aldershot £170; sister Q.A.I.M.N.S., and matron of Military Families Hospitals at stations other than Aldershot, £75; staff nurses and charge nurses £55. These rates will have effect from April 1, 1919. The rates of pay and retired pay of matrons-in-chief and principal matrons are still under consideration.

